# Association between Androgen Deprivation Therapy and Risk of Dementia in Men with Prostate Cancer

**DOI:** 10.3390/cancers13153861

**Published:** 2021-07-31

**Authors:** Jui-Ming Liu, Chin-Yao Shen, Wallis C. Y. Lau, Shih-Chieh Shao, Kenneth K. C. Man, Ren-Jun Hsu, Chun-Te Wu, Edward Chia-Cheng Lai

**Affiliations:** 1 Division of Urology, Department of Surgery, Taoyuan General Hospital, Ministry of Health and Welfare, Taoyuan 33004, Taiwan; mento1218@gmail.com; 2 School of Pharmacy, Institute of Clinical Pharmacy and Pharmaceutical Sciences, College of Medicine, National Cheng Kung University, Tainan 70101, Taiwan; steven_shen0507@hotmail.com (C.-Y.S.); s.c.shao@hotmail.com (S.-C.S.); 3 Research Department of Practice and Policy, UCL School of Pharmacy, London WC1N 1AX, UK; wallis.lau@ucl.ac.uk (W.C.Y.L.); kenneth.man@ucl.ac.uk (K.K.C.M.); 4 Centre for Safe Medication Practice and Research, Department of Pharmacology and Pharmacy, Li Ka Shing Faculty of Medicine, The University of Hong Kong, Hong Kong; 5 Department of Pharmacy, Keelung Chang Gung Memorial Hospital, Keelung 20401, Taiwan; 6 Cancer Research Center, Hualien Tzu Chi Hospital, Buddhist Tzu Chi Medical Foundation, Hualien 97002, Taiwan; hsurnai@gmail.com; 7 College of Medicine, Tzu Chi University, Hualien 97004, Taiwan; 8 Department of Urology, Keelung Chang Gung Memorial Hospital, Keelung 20401, Taiwan; wucgmh@gmail.com

**Keywords:** androgen deprivation therapy, dementia, prostate cancer, multi-database study

## Abstract

**Simple Summary:**

Androgen deprivation therapy (ADT) is the gold standard treatment for advanced prostate cancer and the subsequent risk of dementia remains controversial. Previous studies were limited by small sample sizes, short follow-up times, and racial differences. In this population-based cohort study, we used the National Health Insurance Database of Taiwan and The Health Improvement Network database of the United Kingdom to retrospectively study 129,126 men with prostate cancer in the United Kingdom (UK) and Taiwan. Compared with the ADT-naïve control, patients treated with ADT showed no significant increase in the risk of dementia in both the UK and Taiwan populations. Despite the differences in the populations of the two databases, these results suggest no association between the use of ADT and new-onset dementia.

**Abstract:**

The risk of dementia after androgen deprivation therapy (ADT) in patients with advanced prostate cancer (PCa) remains controversial. This study aimed to evaluate the association between ADT and the incidence of dementia in patients with PCa. We identified patients newly diagnosed with PCa in the National Health Insurance Database of Taiwan from 1 January 2002 to 30 June 2016 and in The Health Improvement Network of the United Kingdom (UK) from 1 January 1998 to 31 March 2018. We classified patients with PCa into ADT and ADT-naïve groups. Propensity score (PS) methods were used to minimize the differences in characteristics between the groups. We performed a Cox proportional hazard model to obtain the adjusted hazard ratio (HR) to compare the incidence of dementia between the groups. Our ADT group comprised 8743 and 73,816 patients in Taiwan and the UK, respectively, which were matched 1:1 to ADT-naïve patients by PS. The incidence rates of dementia in the ADT group were 2.74 versus 3.03 per 1000 person-years in the ADT naïve groups in Taiwan, and 2.81 versus 2.79 per 1000 person-years in the UK. There was no statistical difference between ADT and ADT-naïve groups (adjusted HR: 1.12; 95% confidence interval (CI): 0.87–1.43 in Taiwan and adjusted HR: 1.02; 95% CI: 0.85–1.23 in the UK). We found no association between the incidence of dementia and ADT in patients with advanced PCa in either database. Further studies are warranted to evaluate other possible triggers of incident dementia in patients receiving ADT for advanced PCa.

## 1. Introduction

Androgen deprivation therapy (ADT) has been a mainstay treatment for advanced prostate cancer (PCa) for decades [1,2]. However, ADT can reduce the level of testosterone which has been reported to be associated with a decline of a cognitive function. Moreover, a reduction in androgen may impede the modulation of β-amyloid protein accumulation, which may also affect cognition [3]. This raises concerns over an increased risk of neurodegenerative diseases such as dementia in patients with advanced PCa who receive ADT. Some studies from the United States have reported a range of a 1.17–2.17 times increased risk of dementia in patients receiving ADT for advanced PCa [4,5,6,7]. A study from Korea found a 17% increased risk of dementia in an ADT group compared to an ADT-naïve group [8]. Jhan et al. found that ADT was associated with an 84% increase in the risk of Alzheimer’s disease (AD) in the population of Taiwan [9]. Furthermore, several studies have revealed that ADT can interfere with the cardiovascular system and worsen patients’ dementia [10,11]. The treatment of dementia and related symptoms, such as the use of antidementia drugs and antipsychotics, may also affect patients’ circulatory system, leading to a deteriorating cycle between neurocognitive and cardiovascular events in patients with advanced PCa.

However, some studies based on populations of the United Kingdom (UK), Australia and Sweden did not support the association between ADT and dementia [10,12,13]. The discrepancy in the findings and conclusions of these studies could be partly explained by differences in the ethnicities of patients. Kao LT et al. used the same database as the study by Jhan et al. from Taiwan but reached an inconsistent conclusion [9,14]. This may reflect the fact that the findings were sensitive to variations of study design, outcome definitions and analytic approaches. In particular, compared to ADT-naïve patients, patients on ADT were generally older and more likely to be in a more severe state of PCa, and thus at higher risk of dementia, raising the question of selection bias. Since patients who died could not develop dementia, a competing risk due to mortality may have arisen. On the other hand, since patients who could receive ADT had not died or developed dementia before they received treatment, immortal-time bias in the analysis could not be excluded from the analyses. Taking together all the possible biases in the analysis, the result estimates become unpredictable.

To date, the association between ADT and dementia remains controversial. Possible ethnic influences on the risk of dementia with ADT have not been evaluated. Our study aimed to use two population-based databases, the National Health Insurance Database (NHID) of Taiwan and The Health Improvement Network (THIN) of the UK, to verify the association between ADT and the risk of dementia in patients with PCa, as experienced by different ethnicities. Specifically, we performed a series of analyses including propensity score methods, competing risk models, and landmark methods to address the issues of potential selection bias, competing risk and immortal time bias.

## 2. Materials and Methods

### 2.1. Data Sources

We conducted a retrospective cohort study utilizing the NHID of Taiwan and the THIN database of the UK. The details of the databases are described elsewhere [15,16]. Briefly, the NHID is derived from Taiwan’s National Health Insurance Program which covers 99.9% of the entire population of Taiwan (approximately 23 million individuals). Information on the NHID includes enrollees’ demographics, health care professionals and facilities, service claims from inpatient and ambulatory care, and contracted pharmacies. The THIN contains records from over 800 practices and records of 18 million patients, covering 6.2% of the UK population. Data from THIN are demographically representative of the UK population [15]. THIN contains information such as sociodemographic characteristics, consultations, prescriptions, and diagnoses, and has been used widely in large-scale research studies including those on dementia [17]. Diagnoses in the NHID are coded following the International Classification of Diseases, 9^th^ or 10^th^ revision, Clinical Modification (ICD-9-CM or ICD-10-CM). Diagnoses in THIN are coded according to the READ code. This study was approved by the Institutional Review Board of National Cheng Kung University Hospital (A-ER-107-387) and the Research Ethics Committee for THIN (19THIN084).

### 2.2. Study Population

**Figure 1:**
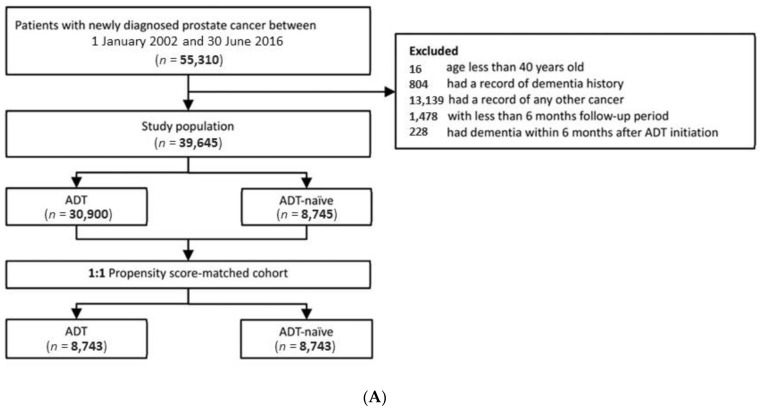

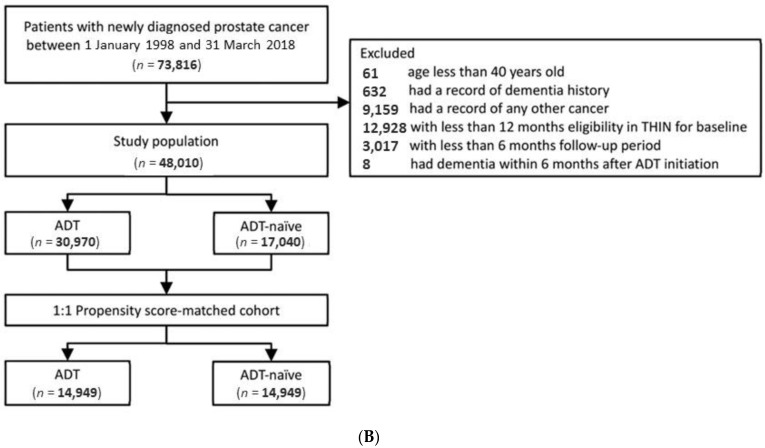
Study cohort selection flowchart: (**A**) the NHID of Taiwan; (**B**) the THIN database of the UK. ADT: androgen deprivation therapy; NHID: National Health Insurance Database; THIN: The Health Improvement Network; UK: United Kingdom.

We included patients newly diagnosed with PCa and recorded in Taiwan’s NHID between 1 January 2002 and 30 June 2016, and those recorded in the UK’s THIN between 1 January 1998 and 31 March 2018. We confirmed the diagnosis of PCa by ICD-9-CM 185 and ICD-10-CM C61 codes in the NHID and by READ codes in the THIN database (Table S1) [18,19]. The index date of each PCa patient in the ADT group was defined as the first date of filling a prescription for ADT. The index date of each patient in the ADT-naïve group was the diagnosis date of PCa. We excluded patients aged 40 years and younger at an index date. Patients who had any record of dementia diagnosis, or any kind of cancer were removed from the analysis. We excluded patients diagnosed with dementia within 6 months after the index date since the occurrence of dementia was unlikely to be related to ADT. We also excluded patients without a minimum of 6 months follow-up to ensure that all study samples had a sufficient observation period. The flowchart of the study population selection is presented in Figure 1A,B.

### 2.3. Exposures and Outcomes

We classified patients into two study groups, the ADT- and ADT-naïve groups according to the records of treatment for advanced PCa. ADT included the use of GnRH agonists (leuprolide, goserelin, triptorelin, and buserelin); oral antiandrogens (cyproterone acetate, bicalutimide, flutamide, and nilutamide); estrogens (diethylstilbestrol and estramustine); and bilateral orchiectomy (Table S2). The study outcome was the incidence of dementia, defined as the first date when the patient received both dementia diagnosis and anti-dementia medication. The diagnosis was defined by the ICD-9 code / ICD-10 code in the NHID (Table S3) and the READ code in the THIN database (Table S4) [17]. The ATC code was used to define anti-dementia agents in both Taiwan and the UK (Tables S3 and S4).

A record of the subsequent use of antidementia drugs was confirmed in order to improve the validity of dementia diagnosis (Table S3). We followed up patients from the index date to the date of diagnosis of dementia, death or the last day of the databases, whichever came first.

### 2.4. Covariates

Baseline covariates were captured covering one year preceding the index date. The covariates consisted of the patients’ age at index date, comorbidities (e.g., hypertension, coronary heart disease, heart failure, atrial fibrillation, peripheral arterial disease, ischemic stroke, diabetes mellitus, chronic obstructive pulmonary disease, chronic kidney disease, chronic liver disease, traumatic brain injury, and depression) and co-medications (e.g., oral hypoglycemic agents, insulin, antiplatelets, anticoagulants, antihypertensive medications, calcium channel blockers, beta-blockers, angiotensin-converting enzyme inhibitors or angiotensin II receptor blockers, non-steroidal anti-inflammatory drugs, cyclooxygenase-2 inhibitors, statins, antidepressants, antipsychotics, and benzodiazepines). 

### 2.5. Statistical Analysis 

We described continuous variables by mean with a standard deviation, and categorical variables by numbers with proportions. We used the standardized mean difference to assess the difference in baseline characteristics between groups. We performed propensity score methods with the matching technique (1:1) to create more comparable groups at the baseline between the ADT and ADT-naïve groups. The propensity score was a predicted probability of being in the ADT group, given the baseline covariates listed in Table 1. Age was included as a categorical variable (<65 y, 65–74 y, 75–84 y, ≥85 y) in the propensity score modelling. We used Kaplan–Meier survival curves along with a log-rank test to compare time to outcome events between groups. We used the Cox proportional hazard model to obtain the adjusted hazard ratios (HR) with a 95% confidence interval (CI) for the comparison of dementia risk between the groups.

### 2.6. Sub-Analysis and Sensitivity Analysis 

Patients were classified into several subgroups by different types of ADT (i.e., GnRH agonist-based treatment or oral antiandrogens only) and by duration of ADT use (<6, 6–12, 13–18, 19–24 and 24+ months) for sub-analysis. We conducted a series of sensitivity analyses to examine the robustness of the results. First, we used the propensity score with inverse probability of treatment weighting (IPTW) and standardized mortality ratio weighting (SMRW) to create homogeneous groups for comparisons. We performed both IPTW and SMRW methods because the estimates from IPTW reflected the average treatment effects in the population, while the SMRW generated average treatment effects in treated patients [20]. Because the study samples could be old and sensitive to a competing risk of mortality, we used cause-specific hazard model and a sub-distribution hazard model to assess the influence from competing effects due to mortality [21]. Moreover, to address the issue of immortal-time bias in the analysis, we used the landmark method with 1- and 2-year landmark periods, to reduce the influence of the bias and to estimate the results [22]. We conducted all analyses using SAS version 9.4 (SAS Institute, Cary, NC, USA).

## 3. Results

We identified a total of 8743 and 73,816 patients in the ADT group, in Taiwan and in the UK, respectively, which matched the same numbers of ADT-naïve patients by PS. The distribution of baseline covariates between the ADT group and ADT naïve groups was similar (SMD < 10%) in both countries after propensity score matching. The mean age was 70.3 years (SD 8.9) in the ADT group and 69.8 (SD 8.9) years in the matched ADT-naïve patients in Taiwan, and 70.0 years (SD 8.5) and 69.2 years (SD 8.7), respectively, in the UK. The rates of diabetes mellitus were 20.6% and 21.7% for ADT and the matched ADT naïve groups, respectively, in Taiwan, but only 10.0% and 10.5%, respectively, in the UK; the rates of benzodiazepine use were 39.1% and 40.7% for the ADT and ADT-naïve groups, respectively, in Taiwan, but only 8.0% and 8.1%, respectively, in the UK. The details of patients’ baseline characteristics are presented in Table 1.

**Figure 2:**
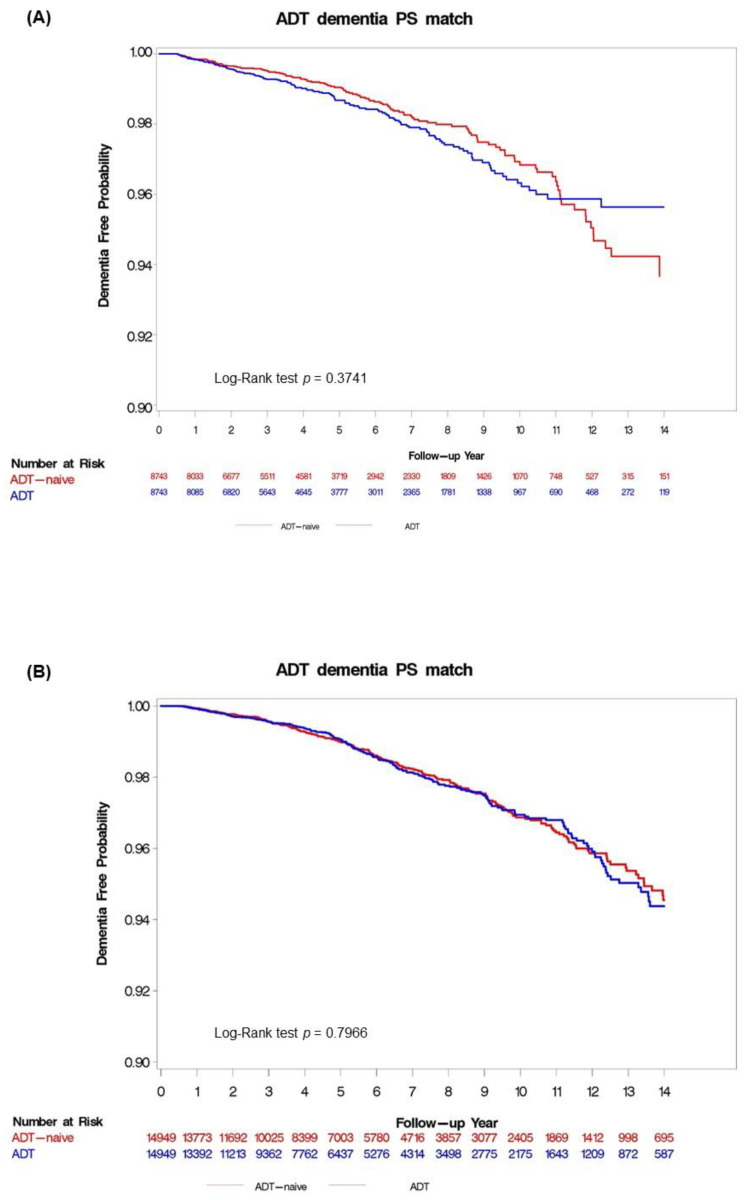
Kaplan–Meier curves according to androgen deprivation therapy (ADT) use for the cumulative probability of remaining dementia-free in the propensity score-matched analysis: (**A**) Taiwan; and (**B**) UK. ADT: androgen deprivation therapy; PS: propensity score; UK: United Kingdom.

The Kaplan–Meier curves for the analyses of Taiwan and the UK are presented in Figure 2A,B, respectively. The overall incidence rates of dementia in the ADT group were 2.74 versus 3.03 per 1000 person–years in the matched ADT-naïve group in Taiwan, and 2.81 versus 2.79 per 1000 person–years, respectively, in the UK. There was no statistically significant difference between the ADT group and the matched ADT-naïve group (adjusted HR: 1.12; 95% CI: 0.87–1.43 in Taiwan and adjusted HR: 1.02; 95% CI: 0.85–1.23 in the UK). The results of subgroup analyses were largely consistent with the main analysis and between countries, as listed in Table 2. There was no difference in the risk of dementia between the ADT and the matched ADT-naïve group within the subgroups of patients receiving GnRH-based therapy (adjusted HR: 0.78; 95% CI: 0.59–1.05 in Taiwan and adjusted HR: 0.99; 95% CI: 0.83–1.2 in the UK) or oral antiandrogens only (adjusted HR: 4.18; 95% CI: 0.93–1.49 in Taiwan and adjusted HR: 1.15; 95% CI: 0.77–1.74 in the UK).

The sensitivity analyses showed results consistent with the main analysis, including the analysis using PS with IPTW (adjusted HR: 0.91; 95% CI: 0.76–1.09 in Taiwan; adjusted HR: 0.99; 95% CI: 0.86–1.14 in the UK) and SMRW (adjusted HR: 1.03; 95% CI: 0.90–1.17 in Taiwan; adjusted HR: 0.98; 95% CI: 0.87–1.10 in the UK). The analyses using a cause-specific hazard model (adjusted HR: 1.12; 95% CI: 0.88–1.43 in Taiwan; adjusted HR: 1.02; 95% CI: 0.85–1.23 in the UK), sub-distribution hazard model (adjusted HR: 0.93; 95% CI: 0.73–1.20 in Taiwan; adjusted HR: 0.85; 95% CI: 0.71–1.02 in the UK), by a 1 y landmark period (adjusted HR: 0.98; 95% CI: 0.74–1.29 in Taiwan; adjusted HR: 1.12; 95% CI: 0.95–1.32 in the UK), and by 2-year landmark analysis (adjusted HR: 1.07; 95% CI: 0.80–1.44 in Taiwan; adjusted HR: 1.00; 95% CI: 0.84–1.20 in the UK) indicated no difference in the incidence rate of dementia between the ADT group and matched ADT-naïve group (Table 3).

## 4. Discussion

In this multi-country population-based study, we evaluated the risk of new-onset dementia subsequent to ADT in 129,126 men with PCa in the UK and Taiwan. Compared to the ADT-naïve control, patients treated with ADT suffered no significant increase in the risk of dementia in both the NHID of Taiwan and the THIN database of the UK. A subgroup analysis showed that a longer duration of ADT did not appear to increase the risk of dementia in either the NHID or THIN databases. Despite the different populations of the two databases, these results suggest no association between the use of ADT and new-onset dementia.

The association between ADT and the incidence of dementia has been discussed for years. Some studies have indicated that the reduced testosterone level [10,13,23] from ADT leads to poor performance in terms of visual memory, verbal memory, and visuospatial rotation in healthy older men [3,24]. However, other studies have failed to deliver any evidence of substantial change in cognitive function after a decrease in testosterone levels [25,26]. A recent meta-analysis showed that ADT use in prostate cancer patients did not increase the risk of cognitive impairment [27]. However, other meta-analysis studies provided inconclusive evidence of cognitive impairment after ADT use [28]. Because dementia is a latent and progressive neurodegenerative disease that is hard to observe, detection bias or misclassification of outcomes in studies are hard to avoid. Compared to the ADT-naïve group, patients who received ADT were more likely to be under observation or have signs or symptoms of dementia detected, since they routinely returned to clinics, which may explain the association found in some studies [4,5,6,7,29]. In our study, we used the diagnosis codes combined with records of the subsequent use of dementia drugs to improve the validity of our analysis. The results may reflect the outcome of overt dementia requiring treatment, while minimizing potential bias due to varying levels of detection of relatively mild dementia. 

Men receiving ADT may experience body weight loss, fatigue, reduced physical activity or increased risk of fracture [30], which may greatly affect the patients’ social networking or mental condition. Although reports have indicated that these side effects could be contributing factors to the outcome of dementia, our study did not find an association between ADT and the incidence of dementia. This may suggest that the side effects did not substantially affect the outcome of dementia. The efficacy of ADT has been demonstrated from RCT and observational studies [31,32], however, the maintenance of the good quality of life and patients’ social network after ADT are also important in patients with advanced PCa. Future investigations into the changes in the quality of life and social behaviors after ADT treatment for advanced PCa patients are warranted, to provide better fundamental information to improve patients’ outcomes. 

Previous studies have provided inconsistent results regarding the association between ADT and dementia in men with advanced PCa [7,10,14,33,34,35,36], possibly because of the differences in study designs, outcome definitions or population ethnicities. Our study was specifically designed to address issues that might affect the analysis. We used propensity score methods to create comparable groups at baseline to minimize selection bias. We defined dementia by diagnosis code combined with the subsequent use of dementia medication to improve the validity of the analysis. Cause-specific hazard and sub-distribution hazard analysis were implemented to address competing risk due to mortality [21]. We adopted landmark methods to minimize immortal time bias [22]. We specifically used two large population-based databases from Taiwan and the UK, composed of different ethnicities to reaffirm the association. It has been reported that patients typically receive ADT for two to three years, based on the guidelines for long-term ADT (28–36 months) in prostate cancer [37]. We found that the rates of patients receiving ADT for more than 24 months were 60.5% in Taiwan but only 31.9% in the UK. To address the possible issue of duration of ADT, we stratified patients by different durations and examined the association with incident dementia. However, we did not find any cumulative dose–response relationship with the dementia risk among patients receiving ADT of various durations. 

There seemed to be a higher prevalence of comorbidities in the Taiwan cohort than the UK cohort. This may be due to actual differences in the disease prevalence between the different ethnicities, or partly the result of differences in the healthcare systems and coding practices between Taiwan and the UK. Taiwan uses a health insurance system, the National Health Insurance (NHI), which covers 99.9% of the 23 million residents of Taiwan [38]. The UK uses a publicly funded, universal healthcare system where over 98% of the UK population are registered with a primary care general practitioner [39]. The NHID of Taiwan is an insurance claims database, while the UK’s THIN is a primary care database, and both are nationally representative. The needs of the different healthcare systems could lead to different coding practices in the databases.

In contrast, we observed a higher prevalence of depression in our UK cohort, while the Taiwanese were more likely to be on antidepressants. In general, Asian countries are reported to have lower prevalence rates for depression—between 2 and 5 percent; compared to Western countries—with a higher prevalence of around 10 to 15 percent [40]. The higher use of antidepressants in Taiwan could be due to antidepressants being prescribed for other indications such as sleep aid and chronic pain in cancer patients. For example, tricyclic antidepressants can be prescribed for chronic pain. Trazodone is the second most commonly used antidepressant in Taiwan, which is most frequently prescribed for insomnia [41]. In addition, trazodone may be prescribed to improve erectile function [42] to treat erectile dysfunction caused by ADT in patients with prostate cancer. Benzodiazepine use is higher in Taiwan compared to other Asian countries, as reported by the Research on Asian Psychotropic Prescription Patterns for Antidepressants database [43]. This could be due to higher insurance coverage, easy access to psychotropic medication, low medication costs, and psychopharmacological prescription traditions in Taiwan. 

Although there were some differences in the patients’ characteristics between the two countries’ populations, we obtained consistent results from both countries, whereby no association between ADT and the incidence of dementia was found. The results were further examined by a series of analyses and showed good robustness. The analysis also showed no cumulative dose relationship and no duration relationship through the use of ADT, with the incidence of dementia. This study included two nationwide databases from Taiwan and the UK, delivering consistent results, and the findings are useful to generalize to both Asian and Western populations.

There were several limitations to this study. First, indicators for the severity of advanced PCa such as PSA levels, clinical stages of prostate cancer, and Gleason scores were not available from the databases; hence, confounding by indication remained possible. Second, indicators for the assessment of cognitive functions such as mini-mental status examination and clinical dementia rating scores were not available. No inference on the association between ADT and different degrees of severity of dementia can be made from this study. Third, because propensity scores were derived based on observed covariates, residual confounders such as patients’ body mass index, lipid profiles and inflammation parameters, which are unmeasurable by the claims database, were not addressed in this study. Due to the study design, the comorbidities and comedications of the UK and Taiwan populations differed on account of their different ethnicities, treatment costs, and health insurance policies, all of which could not be examined. Comparisons between the two countries should be made carefully, because the features of the databases, healthcare systems, cultures and characteristics of the populations vary substantially between countries. Nevertheless, we found similar incidence rates of dementia and consistent study results from both populations.

## 5. Conclusions

The study demonstrated that the treatment of patients with ADT for advanced PCa was not associated with a higher risk of dementia in both UK and Taiwan populations. We did not find any cumulative dose effect between ADT and dementia. Further studies are warranted to evaluate other possible triggers of dementia in patients receiving ADT for advanced PCa.

## Figures and Tables

**Table 1:** Characteristics of the study patients.

	Taiwan			UK		
Covariates	ADT	ADT- Naïve	SMD	ADT	ADT- Naïve	SMD
Number of patients	8743	8743		14,949	14,949	
Age, y (SD)	70.3 (8.9)	69.8 (8.9)	0.06	70.0 (8.5)	69.2 (8.8)	0.10
Age group, y, *n* (%)						
<65	2510 (28.7)	2471 (28.3)	0.06	4525 (30.3)	4484 (30.0)	0.02
65–74	3581 (41.0)	3643 (41.7)		6738 (45.1)	6830 (45.7)	
75–84	2181 (24.9)	2180 (24.9)		2965 (19.8)	2966 (19.8)	
≥85	471 (5.4)	449 (5.1)		721 (4.8)	669 (4.5)	
Comorbidity, *n* (%)						
Hypertension	4597 (52.6)	4528 (51.8)	0.02	5956 (39.8)	6074 (40.6)	−0.02
Coronary heart disease	1659 (19.0)	1700 (19.4)	−0.01	2278 (15.2)	2252 (15.1)	0.005
Heart failure	348 (4.0)	315 (3.6)	0.02	457 (3.1)	489 (3.3)	−0.01
Atrial fibrillation	199 (2.3)	208 (2.4)	−0.01	846 (5.7)	878 (5.9)	−0.01
Peripheral arterial disease	247 (2.8)	255 (2.9)	−0.01	439 (2.9)	454 (3.0)	−0.01
Ischemic stroke	851 (9.7)	868 (9.9)	−0.01	509 (3.4)	498 (3.3)	0.004
Diabetes mellitus	1804 (20.6)	1896 (21.7)	−0.03	1502 (10.0)	1577 (10.5)	−0.02
Chronic obstructive pulmonary disease	1087 (12.4)	1086 (12.4)	<0.001	828 (5.5)	791 (5.3)	0.01
Chronic kidney disease	1037 (11.9)	1054 (12.1)	−0.01	1256 (8.4)	1311 (8.8)	−0.01
Chronic liver disease	1119 (12.8)	1128 (12.9)	<−0.001	252 (1.7)	284 (1.9)	−0.02
Traumatic brain injury	155 (1.8)	139 (1.6)	0.01	143 (1.0)	172 (1.2)	−0.02
Depression	278 (3.2)	301 (3.4)	−0.01	2132 (14.3)	2236 (15.0)	−0.02
Co-medication, *n* (%)						
NSAID	6414 (73.4)	6418 (73.4)	<−0.001	3198 (21.4)	3296 (22.0)	−0.02
Aspirin	2043 (23.4)	2078 (23.8)	−0.01	3532 (23.6)	3530 (23.6)	<0.001
Clopidogrel	317 (3.6)	313 (3.6)	<0.001	383 (2.6)	410 (2.7)	−0.01
COX-2 inhibitor	888 (10.2)	896 (10.2)	<−0.001	229 (1.5)	237 (1.6)	−0.004
Anticoagulant agents	149 (1.7)	150 (1.7)	<−0.001	662 (4.4)	688 (4.6)	−0.01
Statin	1705 (19.5)	1790 (20.5)	−0.02	5027 (33.6)	5136 (34.4)	−0.02
Oral hypoglycemic agents	1463 (16.7)	1531 (17.5)	−0.02	978 (6.5)	1006 (6.7)	−0.01
Insulin	306 (3.5)	326 (3.7)	−0.01	211 (1.4)	228 (1.5)	−0.01
ACEi/ARB	3041 (34.8)	3037 (34.7)	<0.001	4438 (29.7)	4565 (30.5)	−0.02
Antidepressants	1192 (13.6)	1223 (14.0)	−0.01	1528 (10.2)	1582 (10.6)	−0.01
Antipsychotics	729 (8.3)	729 (8.3)	<0.001	437 (2.9)	466 (3.1)	−0.01
Benzodiazepines	3420 (39.1)	3561 (40.7)	−0.03	1189 (8.0)	1212 (8.1)	−0.01
Beta-blocker	2216 (25.3)	2253 (25.8)	−0.01	2530 (16.9)	2567 (17.2)	−0.01
CCB	3265 (37.3)	3210 (36.7)	0.01	3318 (22.2)	3359 (22.5)	−0.01
Follow-up years, y, mean (SD)	4.3 (2.2, 7.3)	4.2 (2.1, 7.2)	----	5.3 (4.0)	5.6 (4.1)	-----

ACEi: angiotensin-converting enzyme inhibitor; ADT: androgen deprivation therapy; ARB: angiotensin receptor blocker; CCB: calcium channel blocker; COX-2 inhibitor: cyclooxygenase-2 inhibitors; NSAID: non-steroidal anti-inflammatory drugs; SD: standard deviation; SMD: standardized mean difference.

**Table 2:** Evaluation of the association between androgen deprivation therapy and the incidence of dementia.

	Taiwan					UK				
	Patients	Events	Follow-Up (Person–Years)	Incidence Rate (per 10^3^ Person–Years)	Adjusted HR (95% CIs)	Patients	Events	Follow-Up (Person–Years)	Incidence Rate (per 10^3^ Person–Years)	Adjusted HR (95% CIs)
**Main analysis**										
ADT-naïve group	8743	121	44,181.7	2.74	Reference	14,949	237	84,331.1	2.81	Reference
ADT group	8743	134	44,291.4	3.03	1.12 (0.87, 1.43)	14,949	220	78,765.1	2.79	1.02 (0.85, 1.23)
**Subgroup Analysis by Type of ADT**										
GnRH agonist-based ADT										
ADT-naïve group	8461	121	42,675	2.84	Reference	14,440	236	81,058.3	2.91	Reference
ADT group	8461	75	36,614.8	2.05	0.78 (0.59, 1.05)	14,440	212	75,733.0	2.80	0.99 (0.83,1.20)
Oral antiandrogens only										
ADT-naïve group	7087	114	35,523.9	3.21	Reference	2921	44	15,909.1	2.77	Reference
ADT group	7087	171	42,947.8	3.98	1.18 (0.93, 1.49)	2921	48	14,827.8	3.24	1.15 (0.77, 1.74)
**Subgroup Analysis by Duration of ADT**										
<6 months										
ADT-naïve group	3792	68	18,420.9	3.69	Reference	10,577	184	57,378.2	3.21	Reference
ADT group	3792	37	12,844.8	2.88	0.88 (0.59, 1.32)	10,577	161	44,899.3	3.59	1.14 (0.93, 1.41)
6–12 months										
ADT-naïve group	2987	55	14,497.9	3.79	Reference	7661	138	39,960.7	3.45	Reference
ADT group	2987	41	9135.0	4.49	1.27 (0.85, 1.91)	7661	117	27,875.2	4.20	1.39 (1.09, 1.79)
13–18 months										
ADT-naïve group	1928	39	10,112.9	3.86	Reference	4040	71	21,181.0	3.35	Reference
ADT group	1928	19	5814.1	3.27	1.08 (0.62, 1.88)	4040	73	19,829.6	3.68	1.25 (0.90, 1.74)
19–24 months										
ADT-naïve group	1807	33	10,056.5	3.28	Reference	2158	39	11,252.8	3.47	Reference
ADT group	1807	23	6775.2	3.40	1.19 (0.70, 2.05)	2158	44	12,301.5	3.58	1.21 (0.78, 1.89)
>24 months										
ADT-naïve group	5287	87	32,687.3	2.66	Reference	4769	87	26,349.6	3.30	Reference
ADT group	5287	42	30,348.2	1.38	0.59 (0.40, 0.85)	4769	108	41,467.0	2.60	0.68 (0.52, 0.91)

ADT: androgen deprivation therapy; CI: confidence interval; GnRH: gonadotropin-releasing hormone; HR: hazard ratio.

**Table 3:** Sensitivity analysis.

	Taiwan					UK				
	Patients	Events	Follow-Up (Person-Years)	Incidence Rate (per 10^3^ Person-Years)	Adjusted HR (95% CIs)	Patients	Events	Follow-Up (Person-Years)	Incidence Rate (per 10^3^ Person-Years)	Adjusted HR (95% CIs)
Analysis by PS with multivariate adjustment										
ADT-naïve group	8745	121	44,190.8	2.74	Reference	17,040	243	97,787.1	2.48	Reference
ADT group	30,900	513	149,436.5	3.43	0.98 (0.80, 1.20)	30,970	525	150,495.2	3.49	1.02 (0.87, 1.19)
Analysis by PS with IPTW										
ADT-naïve group	8780.4	156.4	42,884.6	3.65	Reference	17,199	296	92,189.7	3.21	Reference
ADT group	30,882	494.0	150,845.5	3.27	0.91 (0.76, 1.09)	30,912	484	156,175.6	3.09	0.99 (0.86, 1.14)
Analysis by PS with SMRW										
ADT-naïve group	24,442.8	414.5	121,389.2	3.41	Reference	29,596	575	156,092.1	3.68	Reference
ADT group	30,900	513.0	149,436.5	3.43	1.03 (0.90, 1.17)	30,970	525	150,495.2	3.49	0.98 (0.87, 1.10)
Cause-specific hazard model										
ADT-naïve group	8743	121	44,181.7	2.74	Reference	14,949	237	84,331.1	2.81	Reference
ADT group	8743	134	44,291.4	3.03	1.12 (0.88, 1.43)	14,949	220	78,765.1	2.79	1.02 (0.85, 1.23)
Sub-distribution hazard model										
ADT-naïve group	8743	121	44,181.7	2.74	Reference	14,949	237	84,331.1	2.81	Reference
ADT group	8743	134	44,291.4	3.03	0.93 (0.73, 1.20)	14,949	220	78,765.1	2.79	0.85 (0.71,1.02)
1-year landmark period										
ADT-naïve group	11,903	199	80,477.0	2.47	Reference	15,738	283	91,334.5	3.099	Reference
ADT group	11,903	180	67,640.7	2.66	1.12 (0.95, 1.32)	15,738	275	82,845.1	3.319	1.12 (0.95, 1.32)
2-year landmark period										
ADT-naïve group	9382	161	70,109.1	2.30	Reference	12,885	244	72,094.0	3.38	Reference
ADT group	9382	138	61,166.0	2.26	1.16 (0.93, 1.47)	12,885	218	66,517.8	3.28	1.003 (0.84, 1.20)

ADT: androgen deprivation therapy; CI: confidence interval; HR: hazard ratio; IPTW: inverse-probability-of-treatment weighting; PS: propensity score; SMRW: standardized mortality ratio weighting.

## Data Availability

The datasets used and/or analyzed during the current study are available from the corresponding author upon request.

## Supplementary Materials

The following are available online at https://www.mdpi.com/article/10.3390/cancers13153861/s1, Table S1: The ICD-9 / ICD-10 codes of prostate cancer in Taiwan and READ codes in the UK, Table S2: ATC codes for medicines for androgen deprivation therapy, Table S3: ICD-9 codes, ICD-10 codes and ATC codes for identifying dementia in the Taiwan National Health Insurance Database, Table S4: READ codes and ATC codes for identifying dementia outcomes in the UK Health Improvement Network database.
