# Supplementary material for: Association between Androgen Deprivation Therapy and Risk of Dementia in Men with Prostate Cancer
Source: Cancers (Basel). 2021 Jul 31;13(15):3861. doi: 10.3390/cancers13153861 (PMC8345520; doi:10.3390/cancers13153861)
Supplement: Supplementary file 1 [file cancers-13-03861-s001.zip › cancers-1287180-supplementary.pdf]

cancers MDPI

Supplementary Material: Association between Androgen Deprivation Therapy and Risk of Dementia in Men with Prostate Cancer

Table S1. The ICD-9 / ICD-10 codes of prostate cancer in Taiwan and READ codes in the UK.

**Taiwan**	
**ICD-9 codes**	**Description**
185	Malignant neoplasm of prostate
**ICD-10 codes**	**Description**
C61	Malignant neoplasm of prostate
**UK**	
**READ codes**	**Description**
1427000	H/O: prostate cancer
4M0..00	Gleason grading of prostate cancer
4M00.00	Gleason prostate grade 2-4 (low)
4M01.00	Gleason prostate grade 5-7 (medium)
4M02.00	Gleason prostate grade 8-10 (high)
B46..00	Malignant neoplasm of prostate
B58y500	Secondary malignant neoplasm of prostate
B7C2000	Adenoma of prostate
B834.00	Carcinoma in situ of prostate
B834000	High grade prostatic intraepithelial neoplasia
B834100	Prostatic intraepithelial neoplasia
B915.00	Neoplasm of uncertain behavior of prostate
K223.00	Dysplasia of prostate

Table S2. ATC codes for medicines for androgen deprivation therapy.

ATC codes	Description
**Anti-androgens**	
G03HA01	Cyproterone
G03HB01	Cyproterone And Estrogen
L02BB03	Bicalutamide
L02BB01	Flutamide
**Gonadotropin releasing hormone analogues**	
L02AE02	Leuprorelin
L02AE03	Goserelin
L02AE04	Triptorelin
**Estrogens**	
G03CB02	Diethylstilbestrol
G03CC05	Diethylstilbestrol
L02AA01	Diethylstilbestrol

Table S3. ICD-9 codes, ICD-10 codes and ATC codes for identifying dementia in the Taiwan National Health Insurance Database.

Codes	Description
**ICD-9 codes**	
290	Dementias
294.1	Dementia in conditions classified elsewhere
331.0	Alzheimer's disease
331.1	Frontotemporal dementia
**ICD-10 codes**	
F01	Vascular dementia
F02	Dementia in other diseases classified elsewhere
F03	Unspecified dementia
G30	Alzheimer's disease
G31.0	Frontotemporal dementia
**ATC codes**	
N06DA02	Donepezil
N06DA03	Rivastigmine
N06DA04	Galantamine
N06DA52	Donepezil and memantine
N06DA53	Donepezil, memantine and Ginkgo folium
N06DX01	Memantine

Table S4. READ codes and ATC codes for identifying dementia outcomes in the UK Health Improvement Network database.

READ codes	Description
Eu00.00	[X]Dementia in Alzheimer's disease
Eu00000	[X]Dementia in Alzheimer's disease with early onset
Eu00011	[X]Presenile dementia, Alzheimer's type
Eu00012	[X]Primary degen. dementia, Alzheimer's type, presenile onset
Eu00013	[X]Alzheimer's disease type 2
Eu00100	[X]Dementia in Alzheimer's disease with late onset
Eu00111	[X]Alzheimer's disease type 1
Eu00112	[X]Senile dementia, Alzheimer's type
Eu00113	[X]Primary degen. dementia of Alzheimer's type, senile onset
Eu00200	[X]Dementia in Alzheimer's disease, atypical or mixed type
Eu00z00	[X]Dementia in Alzheimer's disease, unspecified
Eu00z11	[X]Alzheimer's dementia unspecified
F110.00	Alzheimer's disease
F110000	Alzheimer's disease with early onset
F110100	Alzheimer's disease with late onset
Fyu3000	[X]Other Alzheimer's disease
E004.00	Arteriosclerotic dementia
E004.11	Multi infarct dementia
E004000	Uncomplicated arteriosclerotic dementia
E004100	Arteriosclerotic dementia with delirium
E004200	Arteriosclerotic dementia with paranoia
E004300	Arteriosclerotic dementia with depression
E004z00	Arteriosclerotic dementia NOS
Eu01.00	[X]Vascular dementia
Eu01.11	[X]Arteriosclerotic dementia
Eu01000	[X]Vascular dementia of acute onset

Eu01100	[X]Multi-infarct dementia
Eu01111	[X]Predominantly cortical dementia
Eu01200	[X]Subcortical vascular dementia
Eu01300	[X]Mixed cortical and subcortical vascular dementia
Eu01y00	[X]Other vascular dementia
Eu01z00	[X]Vascular dementia, unspecified
E012.00	Other alcoholic dementia
E012.11	Alcoholic dementia NOS
E041.00	Dementia in conditions EC
Eu02.00	[X]Dementia in other diseases classified elsewhere
Eu02000	[X]Dementia in Pick's disease
Eu02100	[X]Dementia in Creutzfeldt-Jakob disease
Eu02200	[X]Dementia in Huntington's disease
Eu02300	[X]Dementia in Parkinson's disease
Eu02400	[X]Dementia in human immune-def. virus [HIV] disease
Eu02y00	[X]Dementia in other specified disease class if elsewhere
Eu10711	[X]Alcoholic dementia NOS
F111.00	Pick's disease
Eu02500	[X]Lewy body dementia
E02y100	Drug-induced dementia
F116.00	Lewy body disease
1461.00	H/O: dementia
66h..00	Dementia monitoring
6AB..00	Dementia annual review
9hD..00	Exception reporting: dementia quality indicators
9hD0.00	Excepted from dementia quality indicators: Patient unsuitable
9hD1.00	Excepted from dementia quality indicators: Informed dissent
9Ou..00	Dementia monitoring administration
9Ou1.00	Dementia monitoring first letter
9Ou2.00	Dementia monitoring second letter
9Ou3.00	Dementia monitoring third letter
9Ou4.00	Dementia monitoring verbal invite
9Ou5.00	Dementia monitoring telephone invite
E00..00	Senile and presenile organic psychotic conditions
E00..11	Senile dementia
E00..12	Senile/presenile dementia
E000.00	Uncomplicated senile dementia
E001.00	Presenile dementia
E001000	Uncomplicated presenile dementia
E001100	Presenile dementia with delirium
E001200	Presenile dementia with paranoia
E001300	Presenile dementia with depression
E001z00	Presenile dementia NOS
E002.00	Senile dementia with depressive or paranoid features
E002000	Senile dementia with paranoia
E002100	Senile dementia with depression
E002z00	Senile dementia with depressive or paranoid features NOS
E003.00	Senile dementia with delirium
Eu02z00	[X] Unspecified dementia
Eu02z11	[X] Presenile dementia NOS
Eu02z13	[X] Primary degenerative dementia NOS

Eu02z14	[X] Senile dementia NOS
Eu02z16	[X] Senile dementia, depressed or paranoid type
Eu04100	[X]Delirium superimposed on dementia
ZS7C500	Language disorder of dementia
ATC codes	
N06DA02	Donepezil
N06DA03	Rivastigmine
N06DA04	Galantamine
N06DX01	Memantine
